# Supplementary material for: Genetically Elevated Selenoprotein S Levels and Risk of Stroke: A Two-Sample Mendelian Randomization Analysis
Source: Int J Mol Sci. 2025 Feb 14;26(4):1652. doi: 10.3390/ijms26041652 (PMC11855697; doi:10.3390/ijms26041652)
Supplement: Supplementary file 1 [file ijms-26-01652-s001.zip › ijms-3471106-supplementary/Figures S1-S7.pdf]

Outcome	IVW.Model	nSNP	OR(95%CI)	P
Ischemic stroke	fixed effect model	15	1.035(1.010 - 1.061)	0.006
Cardioembolic stroke	fixed effect model	15	0.969(0.916 - 1.025)	0.272
Small vessel stroke	fixed effect model	15	1.026(0.969 - 1.086)	0.378
Large artery stroke	fixed effect model	15	1.077(1.004 - 1.156)	0.038

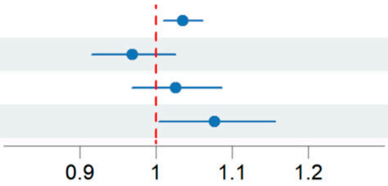

Figure S1. Forest plots for the associations of plasma SELENOS levels with risks of sungroups of ischemic stroke (large artery stroke, cardioembolic stroke and small vessel stroke) in the main inverse-variance weighted Mendelian randomization analysis. OR, odds ratio; 95% CI, 95% confidence interval; SNP, single-nucleotide polymorphism; ICH, intracerebral hemorrhage.

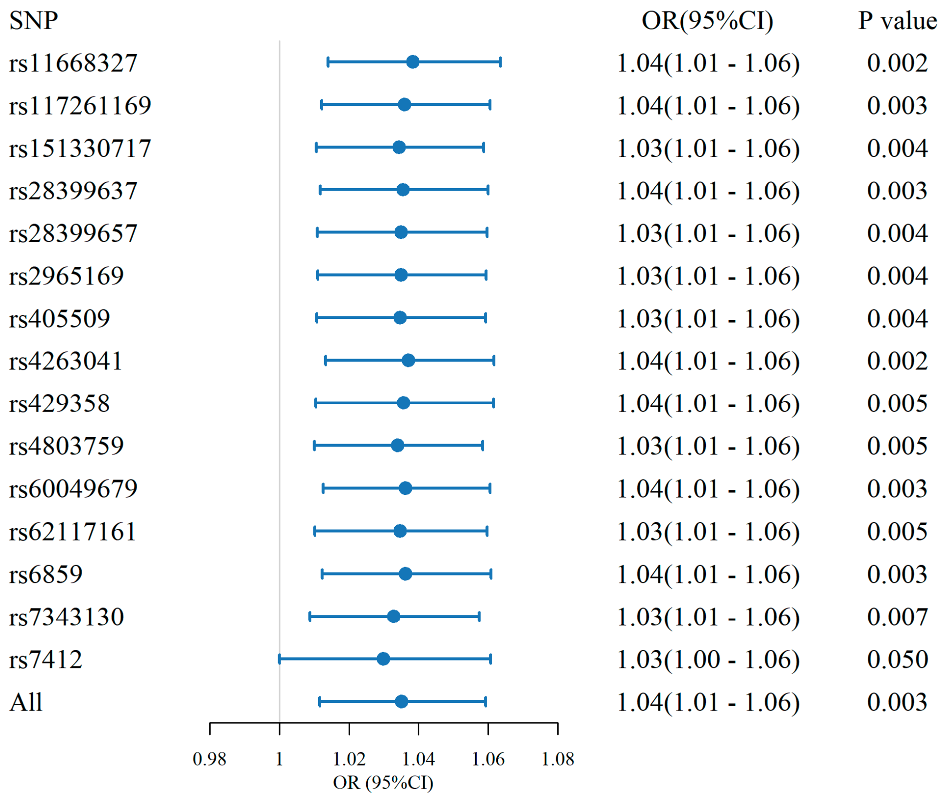

Figure S2. Association between genetically determined plasma SELENOS levels and risk of all-cause stroke in leave-one-out analysis.

SNP, single-nucleotide polymorphism; OR, odds ratio; 95% CI, 95% confidence interval.

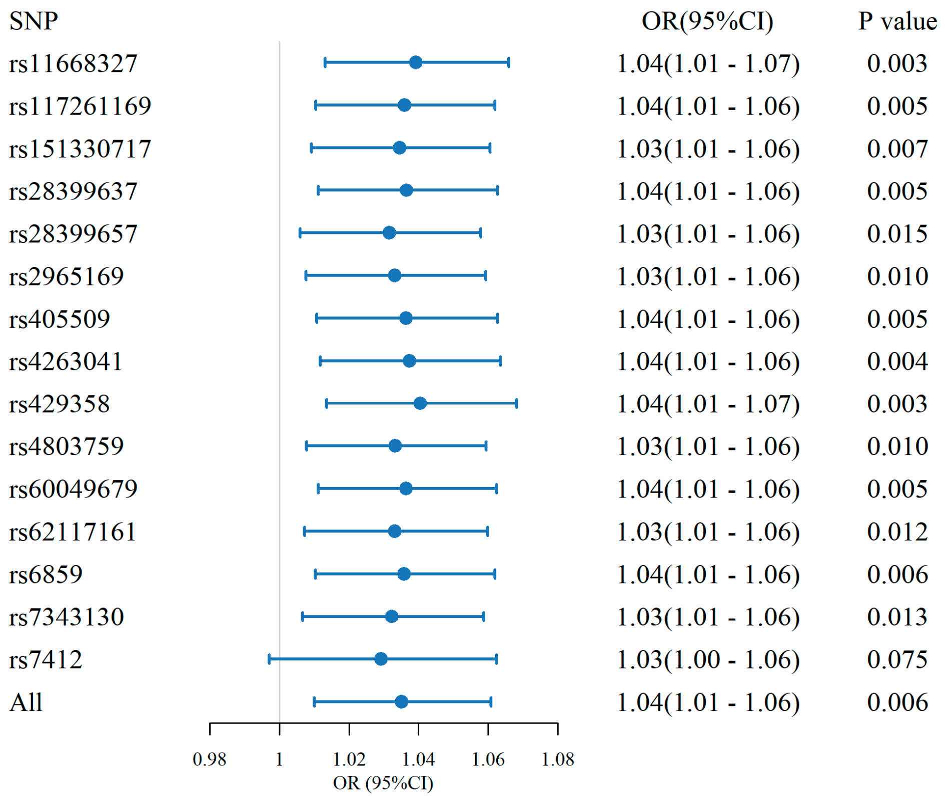

Figure S3. Association between genetically determined plasma SELENOS levels and risk of ischemic stroke (IS) in leave-one-out analysis.

SNP, single-nucleotide polymorphism; OR, odds ratio; 95% CI, 95% confidence interval.

SNP	OR(95%CI)	P value
rs11668327	0.98(0.93 - 1.04)	0.502
rs117261169	0.97(0.92 - 1.03)	0.339
rs151330717	0.97(0.92 - 1.03)	0.358
rs28399637	0.97(0.92 - 1.03)	0.347
rs28399657	0.97(0.91 - 1.02)	0.249
rs2965169	0.97(0.92 - 1.03)	0.337
rs405509	0.97(0.91 - 1.02)	0.241
rs4263041	0.97(0.92 - 1.03)	0.370
rs429358	0.97(0.92 - 1.04)	0.420
rs4803759	0.96(0.91 - 1.01)	0.131
rs60049679	0.97(0.91 - 1.03)	0.259
rs62117161	0.96(0.91 - 1.02)	0.185
rs6859	0.98(0.92 - 1.03)	0.382
rs7343130	0.97(0.91 - 1.02)	0.246
rs7412	0.95(0.88 - 1.01)	0.121
All	0.97(0.92 - 1.03)	0.272

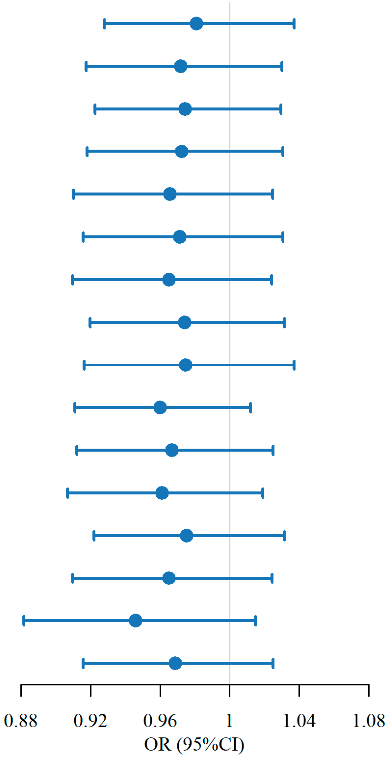

Figure S4. Association between genetically determined plasma SELENOS levels and risk of cardioembolic stroke (CES) in leave-one-out analysis.

SNP, single-nucleotide polymorphism; OR, odds ratio; 95% CI, 95% confidence interval.

SNP	OR(95%CI)	P value
rs11668327	1.03(0.97 - 1.09)	0.381
rs117261169	1.03(0.97 - 1.09)	0.385
rs151330717	1.03(0.98 - 1.09)	0.269
rs28399637	1.03(0.97 - 1.09)	0.365
rs28399657	1.02(0.96 - 1.08)	0.509
rs2965169	1.03(0.97 - 1.09)	0.345
rs405509	1.03(0.97 - 1.09)	0.323
rs4263041	1.03(0.97 - 1.09)	0.289
rs429358	1.03(0.97 - 1.10)	0.342
rs4803759	1.03(0.97 - 1.09)	0.361
rs60049679	1.02(0.97 - 1.08)	0.434
rs62117161	1.03(0.97 - 1.09)	0.334
rs6859	1.02(0.96 - 1.08)	0.500
rs7343130	1.02(0.96 - 1.08)	0.602
rs7412	1.02(0.95 - 1.10)	0.625
All	1.03(0.97 - 1.09)	0.378

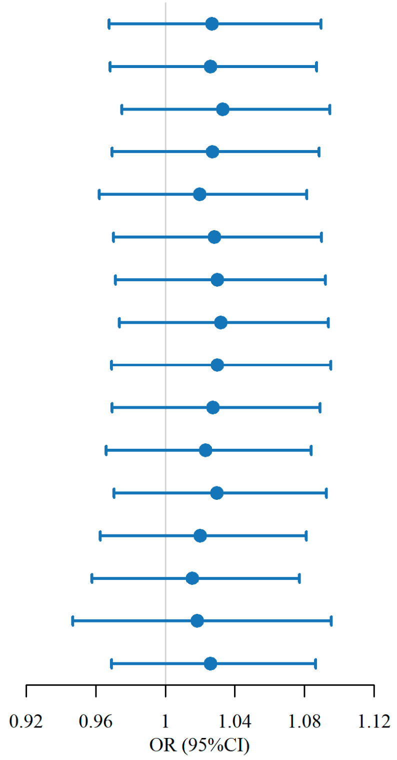

Figure S5. Association between genetically determined plasma SELENOS levels and risk of small vessel stroke (SVS) in leave-one-out analysis.

SNP, single-nucleotide polymorphism; OR, odds ratio; 95% CI, 95% confidence interval.

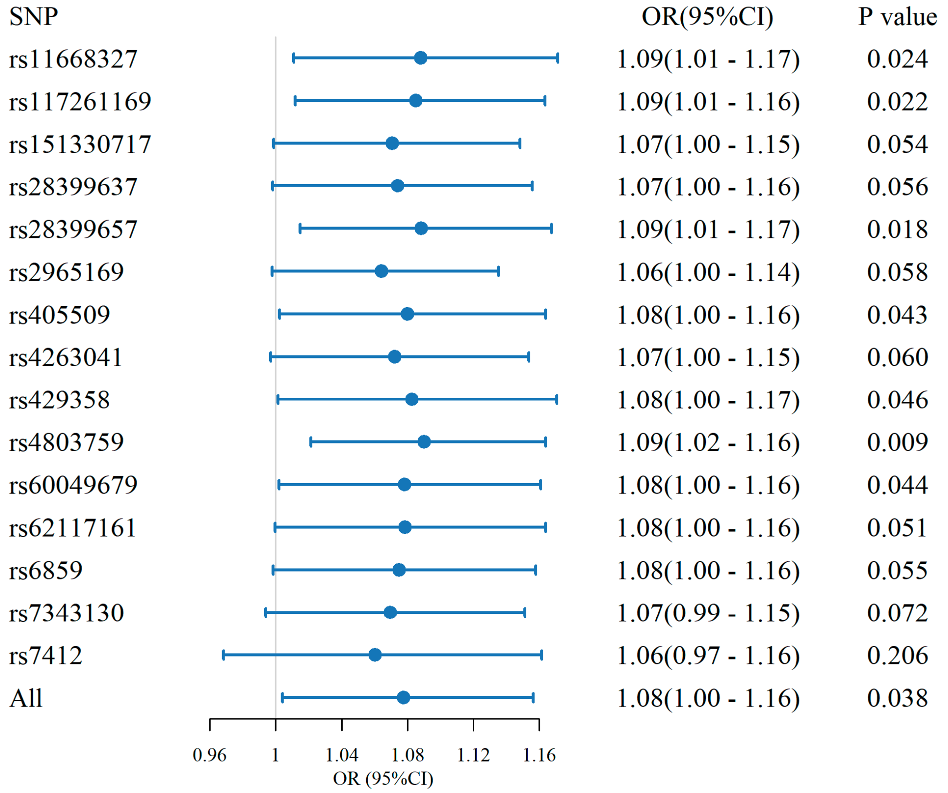

Figure S6. Association between genetically determined plasma SELENOS levels and risk of large artery stroke (LAS) in leave-one-out analysis.

SNP, single-nucleotide polymorphism; OR, odds ratio; 95% CI, 95% confidence interval.

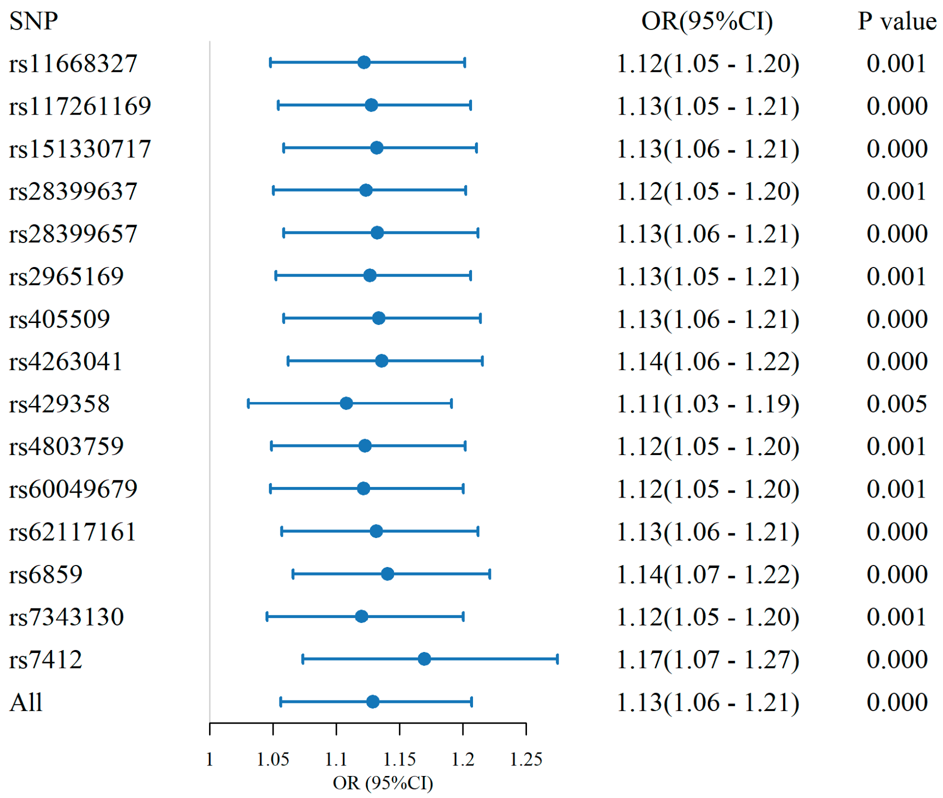

Figure S7. Association between genetically determined plasma SELENOS levels and risk of intracerebral hemorrhage (ICH) in leave-one-out analysis.

SNP, single-nucleotide polymorphism; OR, odds ratio; 95% CI, 95% confidence interval.
