# Supplementary material for: Genetically Elevated Selenoprotein S Levels and Risk of Stroke: A Two-Sample Mendelian Randomization Analysis
Source: Int J Mol Sci. 2025 Feb 14;26(4):1652. doi: 10.3390/ijms26041652 (PMC11855697; doi:10.3390/ijms26041652)
Supplement: Supplementary file 1 [file ijms-26-01652-s001.zip › ijms-3471106-supplementary/Tables S1-S4.pdf]

Table S1. Description of the genetic instruments used in this MR study.

Outcome	Number of SNPs	Variance*	F-statistic†	Power at different ORs and α=0.05		
				1.10	1.20	1.50
All cause stroke	15	36.53	89.99	1.00	1.00	1.00
Ischemic stroke	15	36.53	89.99	1.00	1.00	1.00
Cardioembolic stroke	15	36.53	89.99	1.00	1.00	1.00
Small vessel stroke	15	36.53	89.99	0.99	1.00	1.00
Large artery stroke	15	36.53	89.99	0.98	1.00	1.00
ICH	15	36.53	89.99	0.76	1.00	1.00

ICH, intracerebral hemorrhage;

*Phenotypic variance explained by the genetic instruments used in the present Mendelian randomization analysis.

†Average F-statistic calculated with all included genetic instruments.

Table S2. Results of heterogeneity test and inverse-variance-weighted model used in the analysis.

Outcome	Q value	*P* value	IVW Model
All cause stroke	4.24	0.99	fixed effect model
Ischemic stroke	9.16	0.82	fixed effect model
Cardioembolic stroke	18.59	0.18	fixed effect model
Small vessel stroke	9.24	0.82	fixed effect model
Large artery stroke	17.35	0.24	fixed effect model
ICH	11.67	0.63	fixed effect model

ICH, intracerebral hemorrhage.

Table S3. The results of the Mendelian randomization Steiger test.

Outcome	R^2 for exposure	R^2 for outcome	Correct causal direction	P_{Steiger}
All cause stroke	0.38	2.90×10^{-5}	TRUE	0
Ischemic stroke	0.38	3.81×10^{-5}	TRUE	0
Cardioembolic stroke	0.38	4.89×10^{-5}	TRUE	0
Small vessel stroke	0.38	5.06×10^{-5}	TRUE	0
Large artery stroke	0.38	5.52×10^{-5}	TRUE	0
ICH	0.38	5.15×10^{-5}	TRUE	0

An observed 2-sided $P < 8.33\times10^{-3}$ after Bonferroni correction (0.05/6 [one exposure and six outcomes]) was considered to be statistically significant; ICH, intracerebral hemorrhage.

Table S4. Reverse IVW Mendelian randomization analyses for the associations of all cause stroke, ischemic stroke, ischemic stroke subtypes and ICH with plasma Selenoprotein S levels.

Exposure	NO. SNPs	β(95%CI)	*P* value	Cochran′s Q test	
				Q value	*P* value
All cause stroke	8	0.016(-0.275- 0.308)	0.912	3.32	0.85
Ischemic stroke	9	0.066(-0.186- 0.319)	0.606	4.30	0.83
Cardioembolic stroke	5	-0.112(-0.263- 0.039)	0.145	5.15	0.27
Small vessel stroke	6	-0.040(-0.199- 0.120)	0.626	3.56	0.61
Large artery stroke	4	0.179(0.015- 0.344)	0.033	0.43	0.93
ICH	2	-0.039(-0.388- 0.310)	0.826	2.70	0.10

The SNPs that were identified to be significantly associated with exposures ($P < 5\times10^{-8}$ for all cause stroke , ischemic stroke, cardioembolic stroke, large artery stroke and ICH, $P<5\times10^{-7}$ for small-vessel stroke) and were not in linkage disequilibrium with other SNPs (r2<0.1, with a 10,000 kb clumping window) were selected as genetic instruments for exposures.

An observed 2-sided $P<8.33\times10^{-3}$ after Bonferroni correction (0.05/6 [one exposure and six outcomes]) was considered to be statistically significant.

ICH, intracerebral hemorrhage; IVW, inverse-variance weighted; SNP, single-nucleotide polymorphism; 95% CI, 95% confidence interval.
